# N7-Methylguanosine Genes Related Prognostic Biomarker in Hepatocellular Carcinoma

**DOI:** 10.3389/fgene.2022.918983

**Published:** 2022-06-06

**Authors:** Parbatraj Regmi, Zhi-Qiang He, Thongher Lia, Aliza Paudyal, Fu-Yu Li

**Affiliations:** ^1^ Department of Biliary Surgery, West China Hospital, Sichuan University, Chengdu, China; ^2^ Department of Uro Surgery, West China Hospital, Sichuan University, Chengdu, China; ^3^ Department of Dermatology, West China Hospital, Sichuan University, Chengdu, China

**Keywords:** hepatocellular carcinoma, m7G-related genes, prognosis, nomogram, risk-score

## Abstract

**Background:** About 90% of liver cancer-related deaths are caused by hepatocellular carcinoma (HCC). N7-methylguanosine (m7G) modification is associated with the biological process and regulation of various diseases. To the best of our knowledge, its role in the pathogenesis and prognosis of HCC has not been thoroughly investigated.

**Aim:** To identify N7-methylguanosine (m7G) related prognostic biomarkers in HCC. Furthermore, we also studied the association of m7G–related prognostic gene signature with immune infiltration in HCC.

**Methods:** The TCGA datasets were used as a training and GEO dataset “GSE76427” for validation of the results. Statistical analyses were performed using the R statistical software version 4.1.2.

**Results:** Functional enrichment analysis identified some pathogenesis related to HCC. We identified 3 m7G-related genes (CDK1, ANO1, and PDGFRA) as prognostic biomarkers for HCC. A risk score was calculated from these 3 prognostic m7G-related genes which showed the high-risk group had a significantly poorer prognosis than the low-risk group in both training and validation datasets. The 3- and 5-years overall survival was predicted better with the risk score than the ideal model in the entire cohort in the predictive nomogram. Furthermore, immune checkpoint genes like CTLA4, HAVCR2, LAG3, and TIGT were expressed significantly higher in the high-risk group and the chemotherapy sensitivity analysis showed that the high-risk groups were responsive to sorafenib treatment.

**Conclusion:** These 3 m7G genes related signature model can be used as prognostic biomarkers in HCC and a guide for immunotherapy and chemotherapy response. Future clinical study on this biomarker model is required to verify its clinical implications.

## Introduction

Liver Cancer is the major cause of cancer-related mortality. Hepatocellular carcinoma (HCC) accounts for approximately 90% of liver cancer-related deaths (Global Burden of Disease Cancer et al., 2017; Bray et al., 2018). Despite the improvement in the surveillance program, most of the patients are diagnosed at an advanced stage with unresectable disease (Bruix et al., 2016; Kudo et al., 2016). In recent years, surgical resection, ablation therapies, transarterial chemoembolization, immunotherapy, and systemic chemotherapeutic agents are used in the treatment of early to advanced staged tumors (Llovet et al., 2021). Furthermore, recent advance in biomedical research has led to the identification of molecular pathogenesis, diagnostic biomarkers, and prognostic biomarkers. However, there are no established biomarkers for predicting the response to therapy which could help the clinicians to guide the appropriate treatment strategy for specific patients (Rizzo and Brandi, 2021; Rizzo and Ricci, 2021).

N7-methylguanosine (m7G), methylation on the N7 position of guanosine is a type of positively charged RNA modification in tRNA, rRNA, or internal mRNA regions (Guy and Phizicky, 2014; Sloan et al., 2017; Malbec et al., 2019). Previous studies have shown that more than 100 genes are associated with methylation in several m7G sites (Song et al., 2020; Xia et al., 2021). N7-methylguanosine (m7G) modification is associated with the biological process and regulation of various diseases (Shaheen et al., 2015; Lin et al., 2018; Ma et al., 2021). Furthermore, few recent studies have shown that methylation and mutation at several m7G sites are associated with the molecular pathogenesis of cancer (Chen et al., 2021; Ma et al., 2021). Therefore identification of the m7G-related genes will help in understanding the molecular pathogenesis of cancer and its role in diagnosis, treatment, and prediction of prognosis. The role of m7G-related genes in the pathogenesis and prognosis of HCC, however, has not been studied in greater detail.

Herein, we perform a bioinformatics analysis to identify the association of several m7G-related genes in the molecular pathogenesis of HCC. Furthermore, we aim to construct a prediction model and nomogram to predict prognosis in patients with HCC, its relation with immune infiltration, and chemotherapy sensitivity.

## Materials and Methods

### Hepatocellular Carcinoma Datasets

The RNA sequencing data of the HCC patients, normal tissues, and their corresponding clinical information were obtained from the TCGA database on 30 June 2021. The TCGA datasets were downloaded from the UCSC Xane website [UCSC Xena (xenabrowser.net)]. Furthermore, to increase the reliability of our study, we extracted the RNA sequencing data of HCC patients and normal tissues from a GEO dataset “GSE76427” and used it for validation of the results from the TCGA datasets.

### Identification of Differentially Expressed m7G-Related Genes in HCC

Lists of the m7G-related genes were extracted from the prior studies (Song et al., 2020; Xia et al., 2021). The expression of these m7G-related genes in the TCGA HCC cohorts and normal tissues were identified using the “limma” package in R.

### Functional Enrichment Analysis of Differentially Expressed m7G-Related Genes

The “clusterprofile”, “enrichplot”, “ggplot2”, “GOplot”, and “library (ReactomePA)” packages in R were used to perform Gene Ontology (GO) and Reactome pathway analysis to identify the significant pathways (adj *p* < 0.05) associated with differentially expressed m7G-related genes.

### Development and Validation of PRG Prognostic Model

The “survival” and “glmnet” packages in R were used for the development of a prognostic model. The relevant clinical information of the patients was extracted from the TCGA database and each gene was screened by using the univariate Cox-regression analysis. The least absolute shrinkage and selection operator (LASSO) regression was used to incorporate the m7G-related genes with *p*-value <0.05 in the univariate analysis. Identified genes from the LASSO analysis were further filtered by the multivariate Cox-regression analysis to establish a risk score. A “forestplot” R package was used to create a forest plot for univariate and multivariate outcomes. The Kaplan-Meier survival curve was constructed for each prognostic gene. Lastly, the identified genes from the multivariate analysis were used to construct the risk score using the formula:

Risk score = (expression level of Gene1 ∗ β1 + expression level of Gene2 ∗ β2 +...+expression level of Genen ∗ βn).

The TCGA HCC cohorts were then divided into high- and low-risk groups based on the median risk score and the corresponding Kaplan-Meier analyses were performed comparing these two subgroups. Finally, the AUC was calculated for 3- and 5-year survival in the ROC curves.

### Construction of a Predictive Nomogram

The “survival” “survminer”, and “rms” packages in R were used for the development of the nomogram. Univariate and multivariate cox regression models were used to identify the independent factors affecting the prognosis of HCC patients. Based on the clinicopathologic features of m7G-related genes from multivariate analysis, a cox-proportional hazard (PH) model was used to develop a nomogram to predict the survival probability. The goodness-of-fit test purposed by Schoenfeld was used to test the validity of PH models.

### Immune Infiltration and Chemotherapy Sensitivity Analysis

CYBERSORT tool was used to estimate the expression of 22 different immune cells in HCC patients. We calculated the immune, stromal, and ESTIMATE scores in the high and low-risk groups by using “estimate” package in R. Furthermore, the relation of risk-score with immune checkpoints and immune-suppressive cytokines was also assessed. Furthermore, we checked if the risk model could serve as a potential predictor of chemotherapeutic sensitivity to sorafenib. We converted the gene expression TCGA datasets into half inhibitory concentrations (IC_50_) data matrix of the sorafenib with the “pRRophetic” package and analyzed the IC_50_ difference between the high-risk and low-risk groups.

### Statistical Analyses

Statistical analyses were performed using the R statistical software version 4.1.2. Gene expression levels between the HCC tumor and normal tissue were performed with a single-factor analysis of variance. Categorical variables were compared by using the Pearson chi-square test. Kaplan-Meier analysis was used to compare the survival outcomes. The univariate and multivariate Cox regression analyses were used to evaluate the independent prognostic value of the identified risk model. All the survival outcomes were presented as *p*-values and hazard ratios (HR) with 95% confidence intervals (CI). Mann-Whitney test was used to compare the immune infiltration and pathway activation between the two groups.

## Results

### Identification of the Differentially Expressed m7G-Related Genes in HCC

**FIGURE 1:**
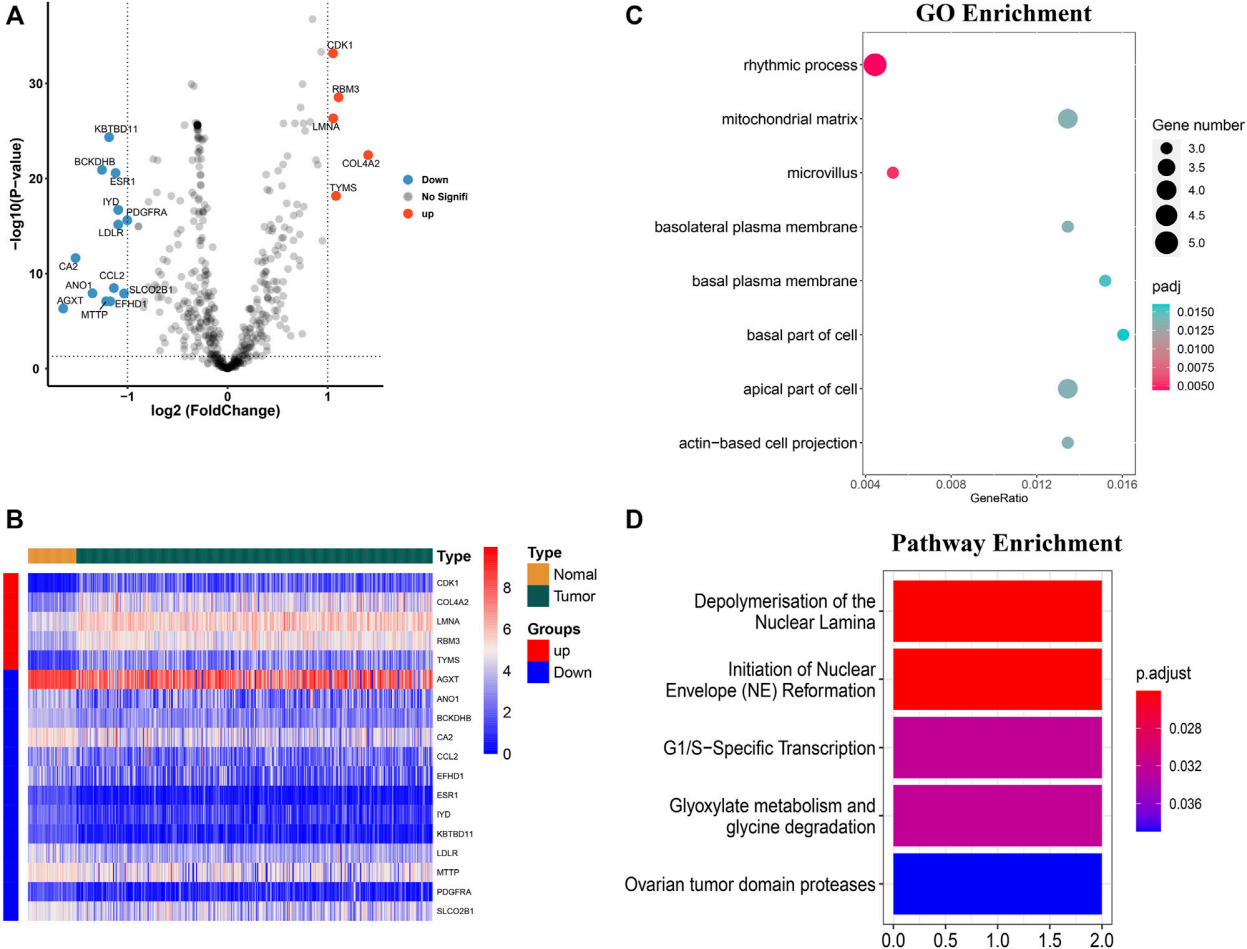
Identification of the differentially expressed m7G-related genes and functional enrichment analysis. **(A)** Volcano plot of the differentially expressed m7g-related genes. **(B)** Heatmap of the differentially expressed m7G-related genes. **(C)** GO functional enrichment analysis. **(D)** Reactome pathway analysis.

A total of 503 M7G-related genes were extracted from the previous studies. Out of them, 492 genes were expressed in HCC and 18 m7G-related genes were differentially expressed (upregulated: 5 and downregulated: 13) in the HCC than in the normal tissues (Figure 1A). Significantly upregulated genes were CDK1, RBM3, LMNA, COL4A2, and TYMS, while the expression of KBTBD11, BCKDHB, ESR1, IYD, PDGFRA, LDLR, CA2, CCL2, ANO1, SLCO2B1, AGXT, EFHD1, and MTTP were downregulated in the tumor. The heatmap of the DEG is shown in Figure 1B.

### Functional Enrichment Analyses of the m7G-Related Genes

GO enrichment analysis of the differentially expressed genes showed that the m7G-related genes were associated with several biological processes, cellular components, and molecular functions. The m7G-related genes were significantly associated with the rhythmic process, microvillus, apical part of the cell, mitochondrial matrix, basolateral plasma membrane, actin-based cell projection, basal plasma membrane, and basal part of the cell (Figure 1C). The Reactome pathway analysis showed that significantly expressed m7G-related genes were associated with depolymerization of the nuclear lamina, initiation of the nuclear envelope reformation, G1/S-specific transcription, glyoxylate metabolism and glycine degradation, and ovarian tumor domain proteases (Figure 1D).

### Construction of m7G-Related Genes Prognostic Model

**FIGURE 2:**
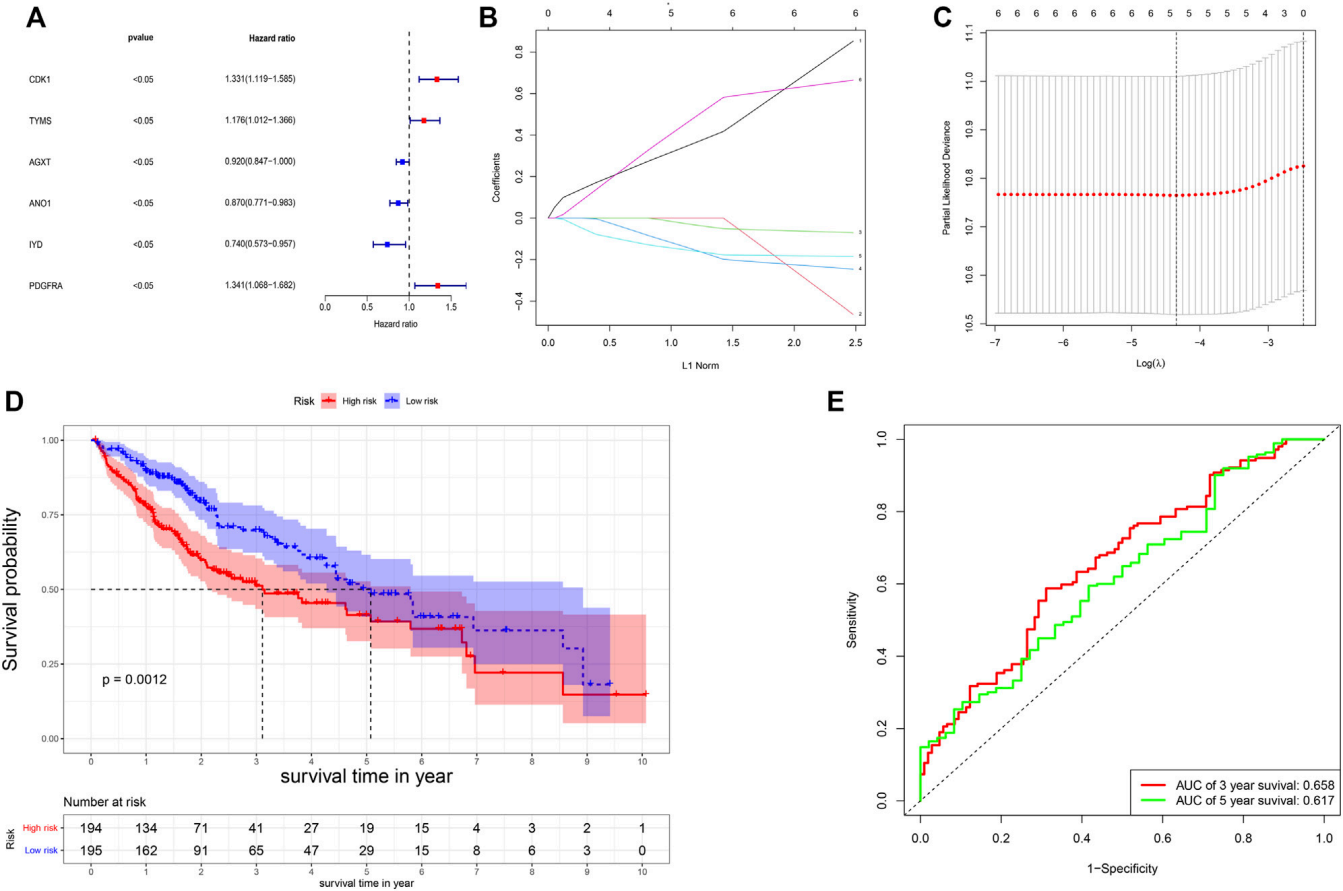
Construction of the risk score. **(A)** Results of the univariate analysis; **(B,C)** Lasso regression analysis of the genes from univariate analysis. **(D)** Survival probability based on the high and low-risk group. **(E)** ROC curve for the 3- and 5-year overall survival.

**FIGURE 3:**
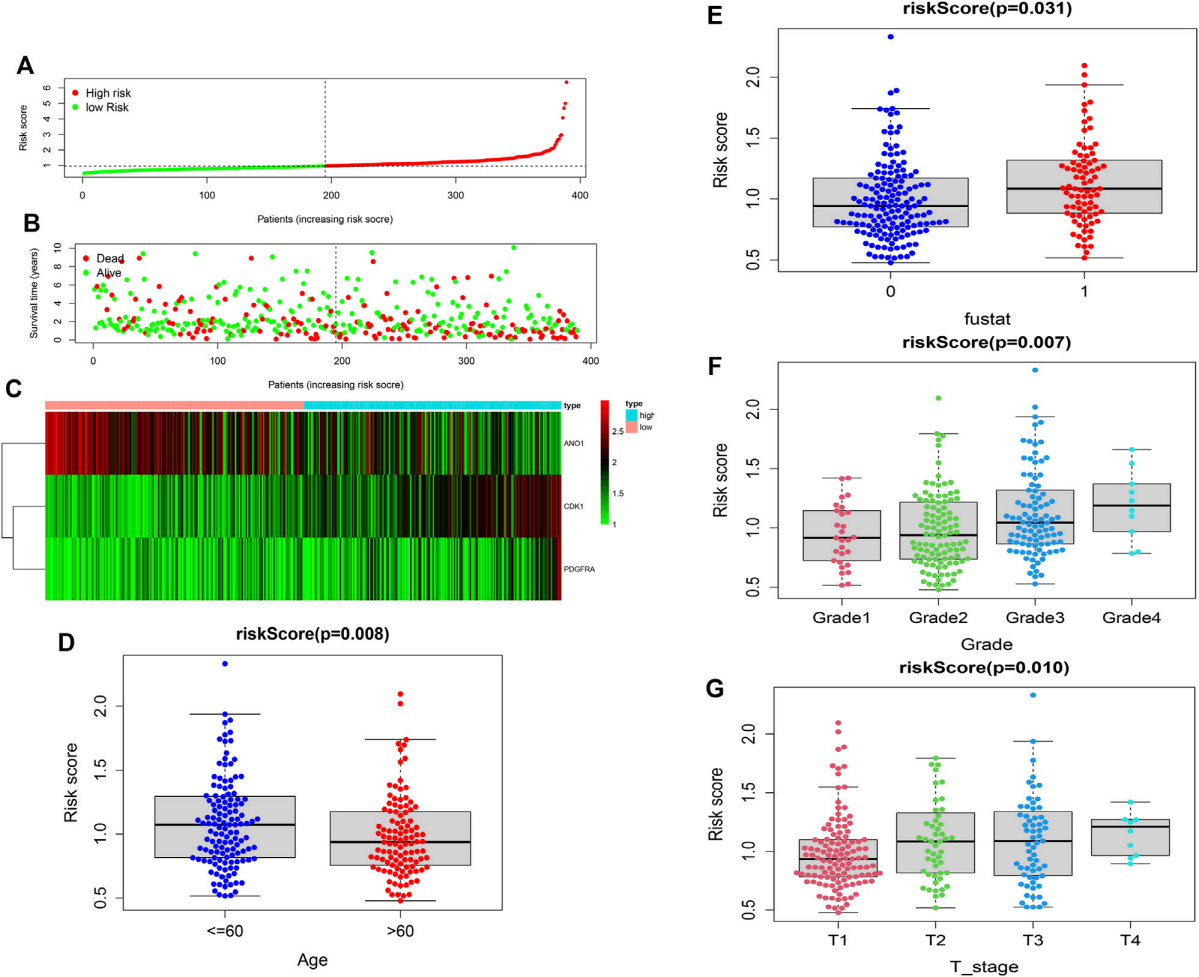
Construction of the risk score. **(A,B)** Distribution of the survival, risk score, and survival of patients with HCC. **(C)** Heatmap of the expression of three m7G-related genes in HCC. **(D)** Relation of the risk score and patient’s age. **(E)** Relation of the risk score with survival status. **(F)** Relation of the risk score with tumor grade. **(G)** Relation of the risk score with T-stage.

The univariate Cox-regression analysis revealed that 6 DEGs (CDK1 (HR: 1.331; 95% CI: 1.119–1.585; *p* < 0.05), TYMS (HR: 1.176; 95% CI: 1.012–1.366; *p* < 0.05), AGXT (HR: 0.920; 95% CI: 0.847–1.000; *p* < 0.05), ANO1 (HR: 0.870; 95% CI: 0.771–0.983; *p* < 0.05), IYD (HR: 0.740; 95% CI: 0.573–0.957; *p* < 0.05), and PDGFRA (HR: 1.341; 95% CI: 1.068–1.682; *p* < 0.05)) were significantly associated with the prognosis of HCC (Figure 2A). Only 5 DEGs (CDK1, AGXT, ANO1, IYD, and PDGFRA) were identified as prognostic genes after LASSO regression analysis (Figures 2B,C). Furthermore, the multivariate Cox-regression analysis identified only 3 DEGs (CDK1, ANO1, and PDGFRA) for the construction of a prognostic model in patients with HCC (Table 1). The patients were then classified into high-risk and low-risk groups based on the median risk score and the Kaplan-Meier survival analysis was performed. The risk score was calculated as: *The Risk score:* Risk score = (0.251) ∗ CDK1 + (−0.122) ∗ ANO1 + (0.318) ∗ PDGFRA. Kaplan-Meier survival curve showed that the prognosis of patients in the high-risk group was significantly poorer than those in the low-risk group (*p* < 0.05) (Figure 2D) with AUCs of 0.658 and 0.617 in 3- and 5-year ROC curves respectively (Figure 2E). As the risk score increased, the risk of death increased but the duration of survival decreased (Figures 3A,B). A Heatmap of the expression of three m7G-related genes in HCC is shown in Figure 3C. Furthermore, we identified that the risk score was significantly associated with the patient’s age (>60 years), survival status, tumor grade, and T-stage (Figures 3D–G).

**TABLE 1:** The multivariate Cox regression analysis for identification of m7g-related genes associated with prognosis in HCC.

Gene	Hazard ratio (HR)	95% confidence interval (CI)	*p*-value
CDK1	1.28485	1.073961 to 1.53715	0.006144
ANO1	0.885348	0.779701 to 1.00531	0.060343
PDGFRA	1.374716	1.112564 to 1.698638	0.003198

### Predictive Nomogram

**FIGURE 4:**
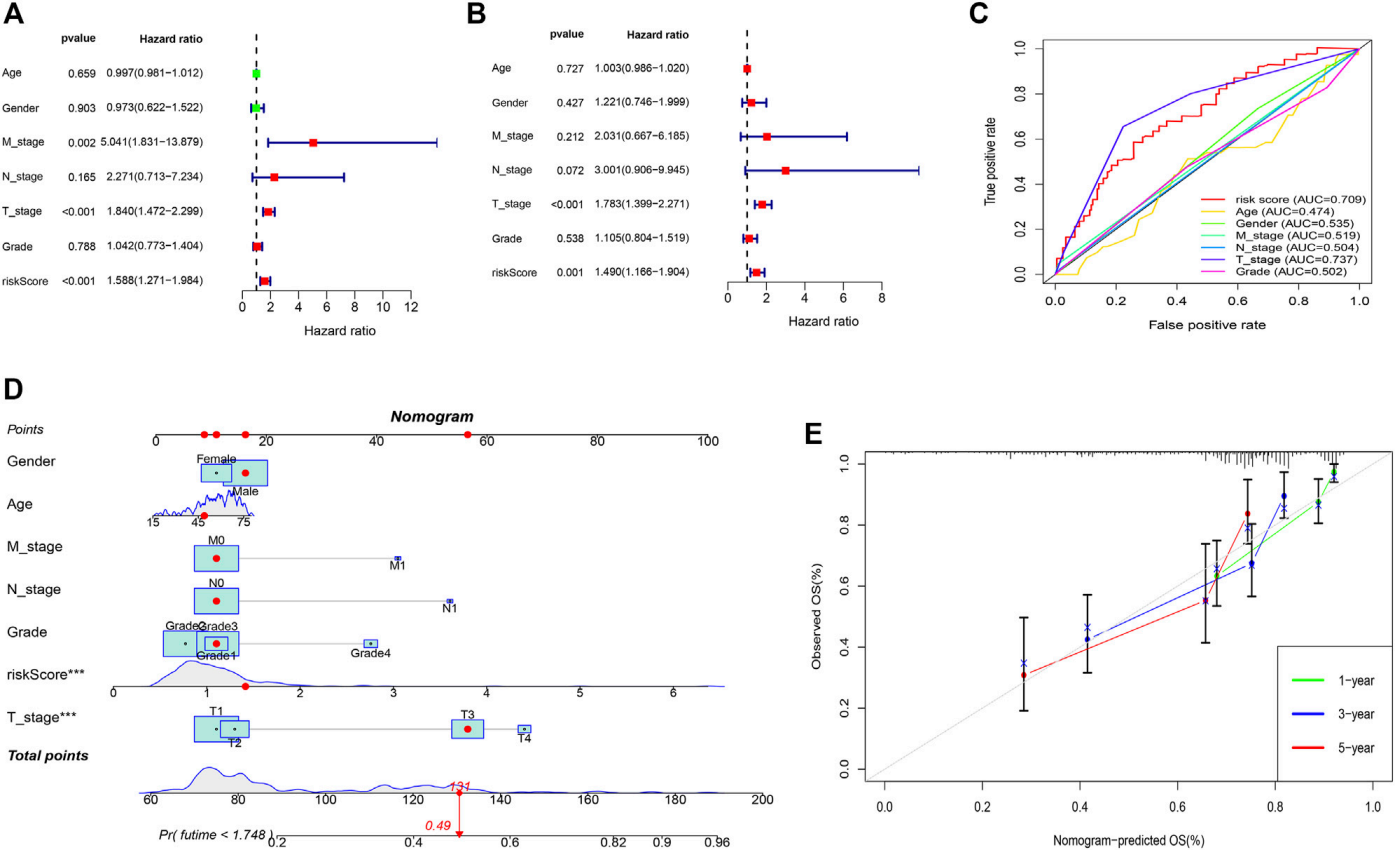
Predictive nomogram. **(A)** Univariate analysis of the clinicopathologic features and the risk score. **(B)** Multivariate analysis of the clinicopathologic features and the risk score. **(C)** ROC curve of the clinicopathologic features and risk score. **(D)** Nomogram to predict the survival of the HCC patients. **(E)** Calibration curve for 1-, 3-, and 5-year survival.

A predictive nomogram was constructed based on the clinicopathologic features and the 3 prognostic m7G-related genes. Results of the univariate showed that the T-stage, M-stage, and risk score were significantly associated with prognosis (Figure 4A). The goodness-of-fit test proposed by Schoenfeld was used to check whether the PH assumption was valid. The Schoenfeld individual test *p* values for risk score age, gender, N-stage, T-stage, M-stage, tumor grade, and risk-score were 0.3689, 0.0605, 0.0896, 0.6864, 0.4132, and 0.8801 respectively. The *p*-value for the global Schoenfeld test was 0.7701 (Supplementary Table S1) (Supplementary Figure S1). The results indicated that all of the factors were satisfied for the PH assumption. Multivariate analysis revealed that only the risk score and T-stage were the independent factors predicting poor prognosis in patients with HCC (Figure 4B). ROC curve of the clinicopathologic features and risk score revealed that the T-stage (AUC of 0.737) and risk score (AUC of 0.709) can better predict the prognosis than other clinicopathologic factors (Figure 4C). The predictive nomogram showed a better prediction of 3- and 5-years overall survival than the other clinicopathological factors (Concordance index: 0.718) (Figure 4D). The calibration curve for 1-, 3-, and 5-year survival showed that the nomogram can accurately predict survival (Figure 4E).

### Association of m7G-Related Genes With Immune Infiltration in the Tumor Microenvironment and Chemotherapy Sensitivity

**FIGURE 5:**
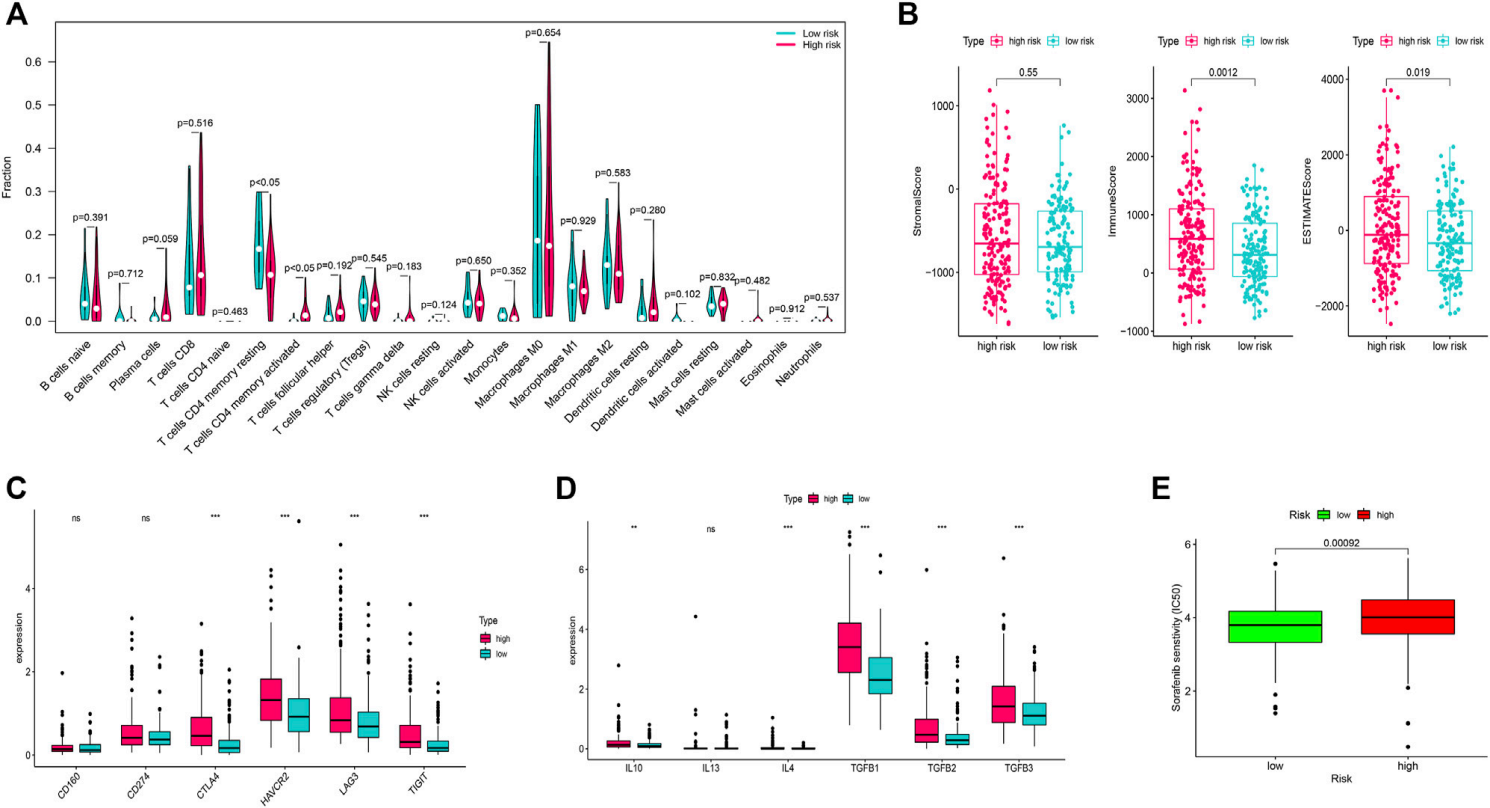
Immune infiltration analysis and chemotherapy sensitivity. **(A)** Relation of the immune cells with a risk score. **(B)** Immune, stromal, and ESTIMATE scores in the high and low-risk groups. **(C)** Relation of the immune checkpoints with risk-score. **(D)**: Relation of the immune-suppressive cytokines with a risk score. **(E)** Sorafenib sensitivity in high-risk and low-risk groups.

We identified that the T cells CD4 memory resting cells were significantly higher in the low-risk group while the T-cells CD4 memory activated cells were higher in the high-risk group (Figure 5A). Furthermore, immune scores and ESTIMATE scores were significantly higher in the high-risk group but there was no significant difference in the stromal score (Figure 5B). Among the several genes involved in immune checkpoints, CTLA4, HAVCR2, LAG3, and TIGT were significantly higher in the high-risk group (Figure 5C). The immune-suppressive cytokines like IL10, IL4, TGFB1, TGFB2, and TGFB3 were also significantly higher in the high-risk group (Figure 5D). We also found that the high-risk group patients were more sensitive to the treatment with sorafenib than the low-risk group (Figure 5E).

### Validation of the Risk Model Accuracy in the GEO Dataset

**FIGURE 6:**
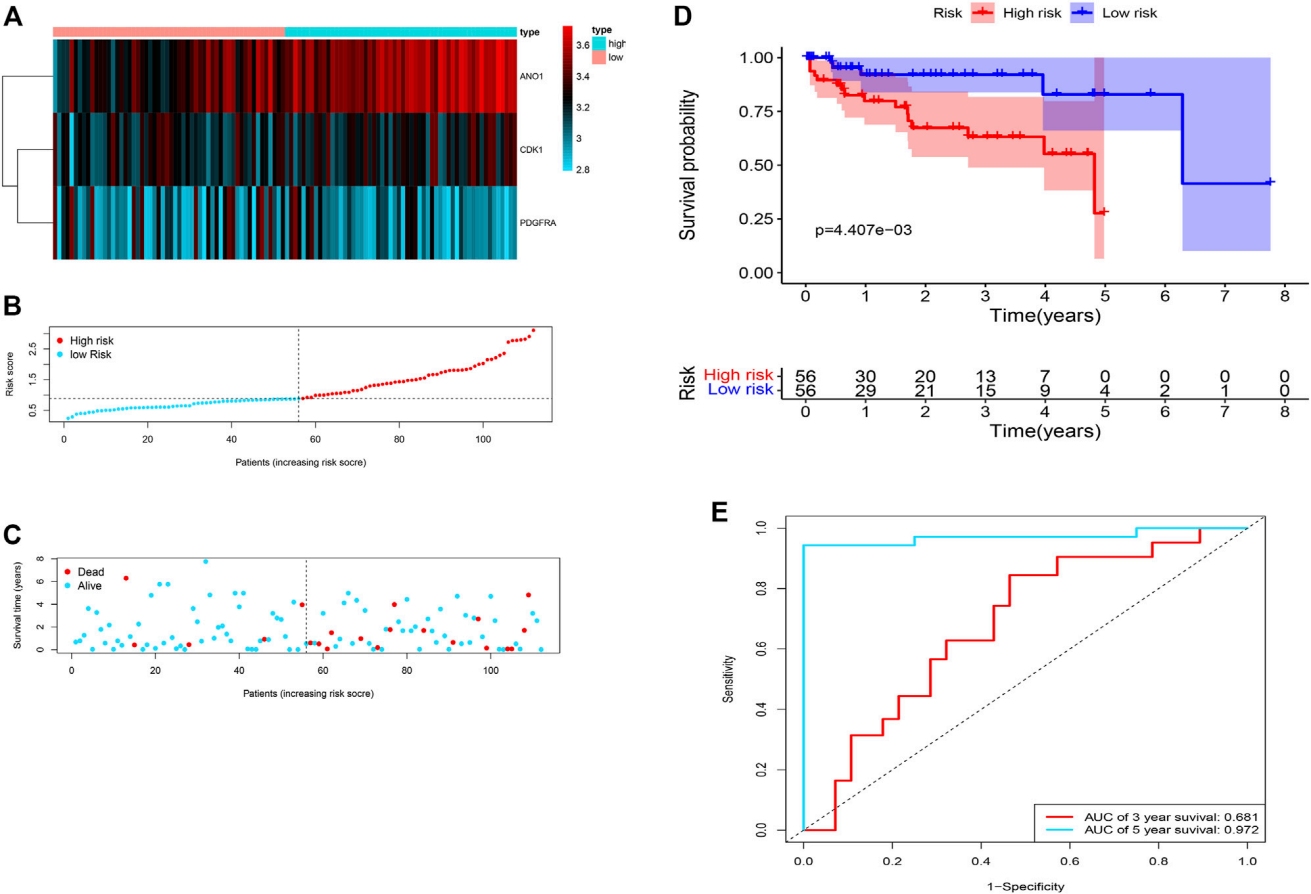
Validation of the risk score in the GEO dataset. **(A)** Heatmap of the 3 differentially expressed m7G-related genes in the validation dataset. **(B,C)** Distribution of the survival, risk score, and survival of patients with HCC. **(D)** Survival probability based on the high and low-risk group. **(E)** ROC curve for the 3- and 5-year overall survival.

The validation of the risk model was performed in the GEO dataset “GSE76427”. The 3 prognostic genes were significantly expressed in the validation dataset (Figure 6A). Similar to the training dataset, as the risk score increased, the risk of death increased but the duration of survival decreased in the validation dataset. (Figures 6B,C). Kaplan-Meier survival curve in the validation dataset showed that the prognosis of patients in the high-risk group was significantly poorer than those in the low-risk group (*p* < 0.001) (Figure 6D) with AUCs of 0.681 and 0.972 in 3- and 5-year ROC curves respectively (Figure 6E).

## Discussion

With the advances in high-throughput sequencing technology, more and more studies have published different kinds of RNA modification including the internal mRNA m7G methylation. In clinical research, several previous studies identified the role of N6-methyladenosine (m6A) remodeling in the molecular pathogenesis of several types of carcinogenesis and the role of m6A-related genes in the prognosis of several types of cancers (Chai et al., 2021; Jin et al., 2021; Han et al., 2022). M6A remodeling has been well studied in HCC as some studies have identified m6A related gene signature model for the prediction of HCC (Jin et al., 2021; Wang et al., 2021). Recently, some studies have shown that some of the m7G-related genes like methyltransferase-like protein-1 (METTL1) and WD repeat domain 4 (WDR4) are involved in the proliferation, migration, and invasion of HCC through m7G tRNA modification (Chen et al., 2021; Xia et al., 2021). However, to our knowledge, the construction of the m7G-related genes prognostic model and its association with immunotherapy in patients with HCC has not been studied in detail.

Our study constructed an m7G associated prognostic model and risk score for predicting the prognosis of HCC which will help in understanding its molecular pathogenesis and will further benefit in understanding the diagnosis, treatment, and prognosis. The results of our study identified that CDK1, ANO1, and PGDFRA-based prognostic models can accurately predict the prognosis in patients with HCC. Mutation in m7G methyltransferase is associated with several diseases like Down syndrome, primordial dwarfism, ovarian carcinogenesis, etc., (Pereira et al., 2009; Shaheen et al., 2015; Trimouille et al., 2018; Xia et al., 2021). Furthermore, it is reported that mutation in m7G sites is also associated with neural line differentiation in embryonic stem cells and it also affects cancer cell viability (Barbieri et al., 2017; Lin et al., 2018).

Cyclin-dependent kinase 1 (CDK1) is highly expressed in HCC tissues and is associated with poor prognosis (Yang et al., 2019; Qiang et al., 2021). It may act as a promising diagnostic, therapeutic, and prognostic marker as well as a promising therapeutic target (Zhou et al., 2019; Yin et al., 2021). Anoctamin 1 (ANO1) regulates cell proliferation during the G (1)/S transition of the cell cycle and is associated with tumorigenesis of several gastrointestinal tumors (Stanich et al., 2011; Zhang et al., 2019; Dong et al., 2021). Overexpression of ANO1 is one of the markers of poor prognosis in HCC (Zhang et al., 2019). Platelet-derived growth factor receptor alpha (PGDFRA) mutation is associated with uncontrolled proliferation and cell growth of gastrointestinal and its inhibition blocks the activity of PDGFRA kinase and improves survival (Kovács et al., 2005; Comandone and Boglione, 2015). PDGFRA is associated with the pathogenesis of hepatic fibrosis during chronic liver injury and is also a prognostic biomarker for HCC (Kikuchi et al., 2017; Yu et al., 2017).

Results of the functional enrichment analysis of the m7G-related genes with GO revealed that some of them were associated with the pathogenesis of HCC. Mitochondrial dysregulation is associated with the aggressiveness of HCC (Min et al., 2021). Actin−mediated cellular activity is associated with cell migration and invasion, which may play important role in the development of cancer (Guan et al., 2020). Proteins like PTEN and aquaporin-9 located in the basal plasma and basolateral membranes have shown some anti-tumorigenic effects against HCC in several preclinical studies (Kato et al., 2020; Liao et al., 2020). These molecules may act as a therapeutic target for the treatment of HCC. Reactome pathway analysis showed that m7G- related genes are associated with nuclear envelope dysregulation (depolymerization and reformation). Nuclear envelope dysregulation and laminopathies are associated with mitogen-activated protein kinase (MAPK) signaling and AKT- mammalian target of rapamycin (mTOR) signaling pathways (Choi and Worman, 2014). These signaling pathways are associated with the pathogenesis of HCC (Pang et al., 2020; Luo et al., 2021). G1 to S transition is a crucial step in HCC proliferation and is negatively associated with prognosis (Zeng et al., 2019). Therefore, identification of the m7G modification sites and their host genes could further help in understanding the pathogenesis of several malignancies and identification of target genes and pathways for the development of newer drugs targeting the m7G sites.

Immune infiltration is also associated with carcinogenesis and the prognosis of HCC (Han et al., 2021; Xu et al., 2021). Moreover, immunotherapy with checkpoint inhibitors has strong anti-tumor activity in HCC (Li et al., 2020; Sangro et al., 2021). Activation of the negative co-stimulatory molecules like cytotoxic T-lymphocyte- associated antigen 4 (CTLA4), LAG3, and TIGIT are associated with the progression of HCC (Moreno-Cubero and Larrubia, 2016; Yu et al., 2021). Clinical studies on anti-CTLA-4 antibodies like tremelimumab and ipilimumab are studying the efficacy and safety with some promising results (Sangro et al., 2013). Therefore, immune checkpoint inhibitors targeting the above-mentioned molecules may be one of the novel therapeutic options for the treatment of HCC. LAG3 is also a predictor of tumor response following therapy for HCC (Guo et al., 2021). We found that the immune-suppressive cytokines like IL10, IL4, TGFB1, TGFB2, and TGFB3 were significantly expressed in patients with high-risk scores. Immune-suppressive cytokines are associated with anti-tumor immune response and the cytokines like IL and TGFB are associated with the pathogenesis of HCC (Zhang et al., 2020; Zhang et al., 2022). Sorafenib is the standard treatment for patients with advanced disease with improvement in survival and radiologic progression of the disease (Llovet et al., 2008). We found that the patients with high-risk scores are more sensitive than those with low-risk scores. This suggests that the developed risk model can not only stratify the prognosis but can also help in identifying the appropriate subgroup of patients for chemotherapy. To our knowledge, there is no single established biomarker for predicting the response to immune-related therapy for the management of advanced HCC. The prognostic marker we developed, with further clinical validation in the future may help the clinicians to guide the appropriate treatment strategy for specific patients.

Several previous studies have shown the reliability of using risk scores for the prediction of the prognosis of HCC. Our risk-score model showed that the patients with high-risk scores had significantly worse survival than those with low-risk scores. The risk score was positively correlated with other well-known prognostic factors like T-stage, N-stage, M-stage, and tumor grade. Furthermore, a quantitative nomogram was developed with the inclusion of risk score and several well-known existing prognostic factors of HCC, and its prognostic ability is better than other existing clinicopathologic factors. This further improved the performance and reliability of the risk score. Therefore, we believe that the proposed model can be used for the stratification of prognosis and identify the appropriate therapies. Lastly, the risk score was used to assess the prognosis in a GEO dataset, which showed that the risk score can accurately predict the prognosis. Therefore, we believe with further validation from future clinical trials, this could be one of the novel prognostic models with higher accuracy.

There are certain limitations to our study. First, *in vivo* and *in vitro* experiments were not performed to verify the results obtained from our study. Second, the results of this study were not validated in the resected specimens of HCC from our institution. Despite these limitations, we believe the results of our study will provide knowledge about the prognostic significance of m7G-related genes in HCC and will certainly help in guiding future clinical studies.

## Conclusion

Our study identified that the 3 m7G-related signature model can be used as a prognostic biomarker in HCC. The functional enrichment analysis of the m7G-related genes showed some of the molecules associated with the pathogenesis and pathways for the progression of HCC. In addition, m7G-related genes were also positively correlated with immune cell infiltration. Future clinical study on this biomarker model is required to verify its clinical implications.

## Data Availability

Publicly available databases were analysed in the study. These datasets can be found here: xenabrowser.net and ncbi.nlm.nih.gov/gds.
