# Supplementary material for: N7-Methylguanosine Genes Related Prognostic Biomarker in Hepatocellular Carcinoma
Source: Front Genet. 2022 Jun 6;13:918983. doi: 10.3389/fgene.2022.918983 (PMC9207530; doi:10.3389/fgene.2022.918983)
Supplement: Supplementary file 1 [file DataSheet1.docx]

**Supplementary materials:**

**Supplementary Figures and tables:**


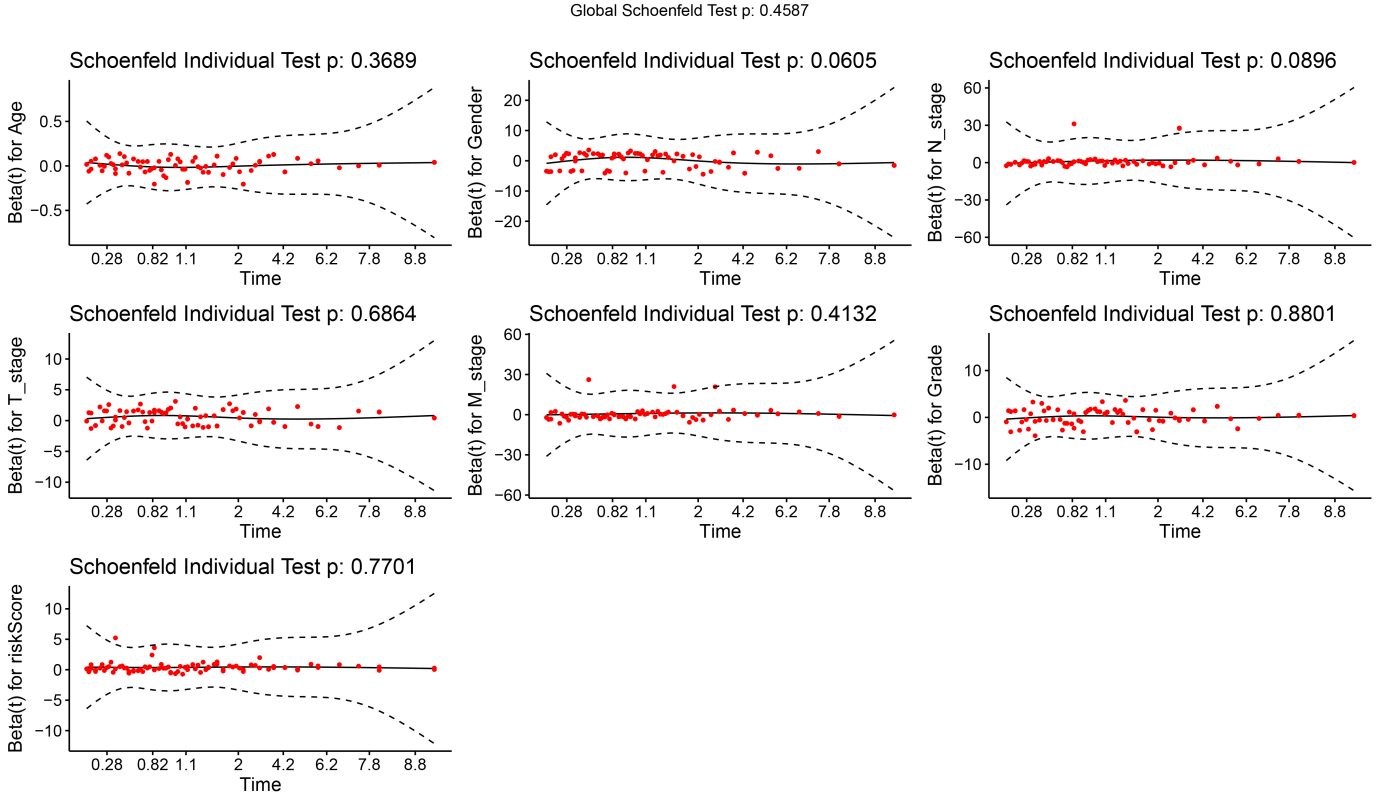


Supplementary Figure 1: Figures showing the results of Schoenfeld test to check the validity of the proportional hazard assumption model.

Supplementary Table 1: The goodness-of-fit test purposed by Schoenfeld was used to check whether the proportional hazard assumption was valid.

| **_Factors_** | **_chi square_** | **_df_** | **_P-value_** |
| --- | --- | --- | --- |
| _Age_ | 0.8072 | 1 | 0.369 |
| _Gender_ | 3.5232 | 1 | 0.061 |
| _N-stage_ | 2.8809 | 1 | 0.090 |
| _T-stage_ | 0.1630 | 1 | 0.686 |
| _M-stage_ | 0.6695 | 1 | 0.413 |
| _Grade_ | 0.0227 | 1 | 0.880 |
| _Risk-score_ | 0.0854 | 1 | 0.770 |
| _Global_ | 6.7192 | 7 | 0.459 |
